# Homeobox and Polycomb target gene methylation in human solid tumors

**DOI:** 10.1038/s41598-024-64569-5

**Published:** 2024-06-17

**Authors:** Reid Blanchett, Kin H. Lau, Gerd P. Pfeifer

**Affiliations:** 1 Department of Epigenetics, Van Andel Institute, 333 Bostwick Ave. NE, Grand Rapids, MI 49503 USA; 2 Bioinformatics and Biostatistics Core, Van Andel Institute, Grand Rapids, MI USA

**Keywords:** Tumour biomarkers, Epigenetics, Epigenomics

## Abstract

DNA methylation is an epigenetic mark that plays an important role in defining cancer phenotypes, with global hypomethylation and focal hypermethylation at CpG islands observed in tumors. These methylation marks can also be used to define tumor types and provide an avenue for biomarker identification. The homeobox gene class is one that has potential for this use, as well as other genes that are Polycomb Repressive Complex 2 targets. To begin to unravel this relationship, we performed a pan-cancer DNA methylation analysis using sixteen Illumina HM450k array datasets from TCGA, delving into cancer-specific qualities and commonalities between tumor types with a focus on homeobox genes. Our comparisons of tumor to normal samples suggest that homeobox genes commonly harbor significant hypermethylated differentially methylated regions. We identified two homeobox genes, *HOXA3* and *HOXD10*, that are hypermethylated in all 16 cancer types. Furthermore, we identified several potential homeobox gene biomarkers from our analysis that are uniquely methylated in only one tumor type and that could be used as screening tools in the future. Overall, our study demonstrates unique patterns of DNA methylation in multiple tumor types and expands on the interplay between the homeobox gene class and oncogenesis.

## Introduction

Epigenetics has become a key component of cancer research. Its dynamic and reversible nature gives the field the potential to produce targets for biomarker identification, cancer therapeutics and interventions. DNA methylation is one such example of an epigenetic modification that plays a large role in oncogenesis. Outside of cancer, genomic imprinting (reviewed in Elhamamsy^1^), cell identity establishment (reviewed in Bogdanovic and Lister^2^), and X chromosome inactivation^3^ are all examples of DNA methylation involvement in essential biological processes. Through the action of DNA methyltransferase enzymes, a methyl group is added to a cytosine in the genomic DNA, preferentially at CpG dinucleotide sequences. In cancers, the DNA hypermethylation is typically focused at CpG islands, which are stretches of CpG-rich DNA, often localized near a gene’s promoter region. The study of DNA hyper- and hypomethylation has led to specific tumor characterization in many different cancers that allow them to be distinguished from healthy tissue or their downstream metastases, aiding in both diagnosis and treatment. The hypermethylation of CpG islands, for example, is a common occurrence in cancers and is in some cases associated with gene inactivation and regulation of tumor suppressor genes^4^.

One such group of differentially methylated genes seen in tumors are the homeobox genes which are defined by the presence of a homeodomain in the encoded proteins, a highly-conserved 60 amino acid domain that binds to DNA ^5^. Many of the resulting homeodomain proteins function as transcription factors^6,7^. In cancer, homeobox genes can be considered “tumor modulators” for their participation as both oncogenes and tumor suppressor genes depending on the context of their expression ^8^. The methylation of CpG islands is the most common manner in which homeobox genes are silenced in solid tumors ^9^, and the resulting homeobox gene dysregulation is generally tissue- and site-specific^10^.

An important layer of regulation of homeobox genes includes the Polycomb Repressive Complex 2 (PRC2), a chromatin-associated methyltransferase complex that can tri-methylate lysine 27 on histone H3 (H3K27), forming H3K27me3, which epigenetically silences the chromatin. Many genes targeted by Polycomb are essential for fundamental, evolutionarily conserved processes such as development and stem cell pluripotency, and commonly encode transcription factors^11^. PRC2 targets also play a role in cancer onset and progression. These target genes have been shown to overlap with differentially methylated CpGs in several human cancers^12–17^.

Though the PRC2 regulates homeobox genes during development, components of the PRC2 can be overexpressed in solid tumors ^18,19^, creating the potential for homeobox gene dysregulation. During tumorigenesis homeobox genes are preferentially targeted for aberrant DNA methylation^13,16,20^. DNA and histone methylation have been shown to cooperate within the epigenomic landscape with a molecular crosstalk that results in an overlap of epigenetic profiles in human disease ^21^. This creates unique patterns in the genome that have the potential to function as biomarkers and treatment targets for cancers driven by DNA methylation aberrations.

Though DNA methylation analyses have led to the characterization of epigenetic markers in many individual tumor types ^22–24^, pan-cancer analyses are able to show both differences and commonalities between tumors. This has the potential to provide biomarkers and targets for individual cancers as well as overall similarities between tumors that could be used to classify and treat cancer as a disease in general. Currently, the DNA methylation landscape has been utilized to produce lists of single hyper- and hypomethylated homeobox genes associated with different tumor types, as reported by Rodrigues et al.^25^. However, a gap exists in the exploration of the intersection between tumor profiles concerning all differentially methylated homeobox genes. Further, the relationship between this distinctly methylated gene class in the context of being PRC2 target genes in a pan-cancer context has never been assessed. The aspect of PRC2 is important because of the intense crosstalk between DNA methylation and histone modifications. Investigation of this extra layer of regulation creates a more complete picture of homeobox dysregulation in tumor tissue and the mechanisms that influence it.

The current study utilizes sixteen TCGA tumor datasets containing Illumina HM450k array methylation data. Our main objective was to determine the general landscape of differentially methylated regions between the cancer types, before narrowing down to investigate the hypermethylation of homeobox genes across the tumors and exploring the role of PRC2 target genes and its intersection with the homeobox gene class. We addressed this objective bioinformatically using already established pipelines for Illumina HM450k array datasets and open-source computational tools. Based on the literature summarized above, we hypothesized that each tumor type, when compared to a set of its normal tissues, would have a majority of hypermethylated differentially methylated regions (DMRs) as defined by our pipeline. Additionally, we expected homeobox genes to display this DMR hypermethylation and exist as a subset of PRC2 target sites. Our results have the potential to produce new methylation biomarkers and broaden our understanding of the role of homeobox genes in oncogenesis as well as the interplay between PRC2 and this specialized gene family.

## Methods

### Definition of differentially methylated regions

All calculations and data processing were done using R version 4.3.0^26^. The data were downloaded from TCGA Genomic Data Commons Portal (GDC) using TCGABiolinks^27–29^, a Bioconductor package. Cohorts used in the analysis include the solid tumor TCGA-BLCA, -BRCA, -CESC, -CHOL, -COAD, -ESCA, -HNSC, -KIRC, -KIRP, -LIHC, -LUAD, -LUSC, -PAAD, -PRAD, -THCA, and -UCEC datasets, with dataset tissue availability in Table 1. The Illumina HM450 bead chip array^30^ data were queried from TCGA GDC and the corresponding calculated beta values for the CpG sites were used for the DMR calculation. Data were restricted to primary solid tumors and normal solid tissue, and all used the hg38 assembly. The SeSAMe package^31–34^ was used for DMR analyses. To define DMRs, the SeSAMe package uses Euclidean distance to group CpGs into segments, and then combines the p-values using Stouffer’s Z-score method with the default value being 0.5. The DMR cutoff is determined empirically using the beta value distribution and a SeSAMe-specific parameter called seg.per.locus which quantifies the expected number of DMRs. Higher values lead to more segments.

**Table 1:** Sample numbers for primary tumor and normal tissues for each TCGA dataset.

TCGA dataset	Primary tumor (*n*)	Solid tissue, normal (*n*)
BLCA	418	21
BRCA	793	97
CESC	307	3
CHOL	36	9
COAD	312	38
ESCA_A	98	16
ESCA_SC	90	16
HNSC	528	50
KIRC	324	160
KIRP	275	45
LIHC	377	50
LUAD	473	32
LUSC	370	42
PAAD	184	10
PRAD	502	50
THCA	507	56
UCEC	438	46

TCGA-ESCA was split into esophageal adenocarcinoma (ESCA_A) and esophageal squamous cell carcinoma (ESCA_SC) due to the presence of equal sample sizes for the tumors and different epigenetic profiles for both cancer subtypes.

After the definition of the differentially methylated regions, the SeSAMe package runs a Benjamini–Hochberg false discovery rate correction. A threshold was set to only use DMRs with an adjusted p value of 0.01 or less. The DMRs were further filtered by taking only the DMRs composed of two or more probes, where each probe represents a CpG site. This 2-loci definition was made to increase confidence in the relevance of the DMR. The list of DMRs was then converted to a GenomicRanges object using the package GenomicRanges^35^. The total number of DMRs was calculated by taking the number of distinct segment IDs. Hypermethylated DMRs were defined as those with a segment estimate greater than zero, while hypomethylated DMRs had a segment estimate less than zero. Using the “stats” R package, a chi-squared test was performed to determine if the hypo- and hypermethylation frequencies were dependent on cancer type.

### Differentially methylated regions overlapping homeobox genes

The GENCODE v44 annotation, restricted to annotated genes, was used in R to find the genomic positions of a list of 206 homeobox genes obtained from Holland et al.^36^. This list was converted to a GenomicRanges object. An extra 10 kb was added to each end of the ranges for the homeobox genes and then using SubsetByOverlaps, the DMR GenomicRanges object was cross referenced with the homeobox GenomicRanges object looking for the genomic regions that overlapped. The number of DMRs encompassing homeobox genes was calculated by taking the number of distinct segment IDs, with the hypermethylated segments having a segment estimate greater than zero. Using the “stats” R package, a chi-squared test was performed to determine if the numbers of hypo- and hypermethylated DMRs overlapping homeobox genes were dependent on cancer type. Additionally, a permutation test was run using the regioneR^37^ package in R to determine whether the number of overlaps between differentially methylated regions and homeobox genes was statistically significant.

### Differentially methylated regions overlapping PRC2 target genes

A similar procedure was followed for a list of PRC2 target genes. First, the human embryonic stem cell H1 cells (hESC-01) reference epigenome for ChIP-Seq analysis of H3K27me3 was downloaded (GSM466734). The use of embryonic stem cells for H3K27me3 was chosen because the majority of regions occupied by PRC2 in embryonic stem cells retain the H3K27me3 mark across differentiated cell types of all lineages, giving a general background for all tissue types used in this study^38^. The processed ChIP-Seq data was used in the form of a .bed file to indicate peak locations. Because the data used the GRch37/hg19 genome assembly, which differs from the TCGA data, the UCSC Lift Genome Annotations tool was used to convert the hESC-01 data to the hg38 assembly. SubsetByOverlaps was then used to find any overlap between the DMR GenomicRanges object and the hESC-01 GenomicRanges object.

This process was then repeated for each of the specific non-cancerous tissue samples that matched a TCGA tissue type. Breast, liver, lung, pancreatic, esophageal, uterine, and thyroid tissue H3K27me3 data were downloaded from ENCODE^39,40^ (https://www.encodeproject.org; Table 2). All data used the hg38 genome assembly.

**Table 2:** Tissue types and experiment IDs for H3K27me3 ChIP-Seq data from ENCODE.

TCGA tissue	Origin of sample	ENCODE experiment
Breast	Breast epithelium	ENCSR134LLK
Esophagus	Esophagus squamous epithelium	ENCSR024JJL, ENCSR952BJX, ENCSR049FUB, ENCSR057BFO
Liver	Hepatocytes	ENCSR637RLN
	Liver, bulk tissue	ENCSR810PYW, ENCSR731AIB
Lung	Lung, bulk tissue	ENCSR800MYV, ENCSR975GDL
Pancreas	Pancreas, bulk tissue	ENCSR133RZO, ENCSR179GVP, ENCSR499GKR, ENCSR511LIV
Thyroid	Thyroid, bulk tissue	ENCSR586DVD
Uterus	Uterus, bulk tissue	ENCSR111DTF, ENCSR839SAN

The ENCODE data was then used to define lists of tissue specific Polycomb target genes. These lists were then intersected with the TCGA DNA methylation data as described above for the homeobox genes. From this analysis, we derived the lists of hypermethylated and hypomethylated Polycomb target genes in a cancer-type-specific manner. Finally, a permutation test was performed to determine whether homeobox genes statistically overlap more H3K27me3 than by random chance. The regioneR package in R was used for the permutation test^37^.

## Results

### Differentially methylated regions in TCGA cancer datasets

**Figure 1:**
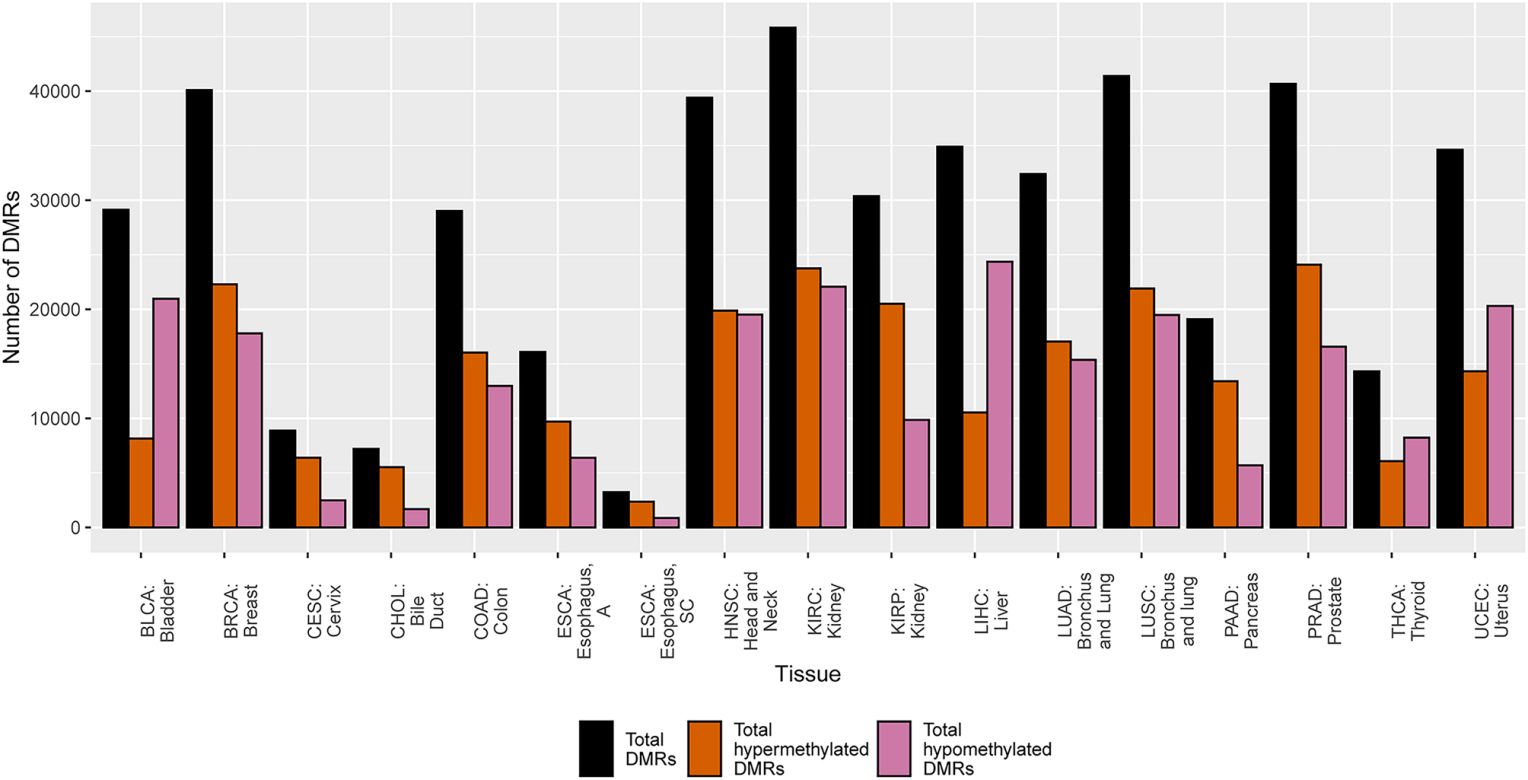
Total hyper- and hypomethylated differentially methylated regions in the listed TCGA cancer type compared to total DMRs.

To characterize the overall DNA methylation landscape of different cancers and create a baseline profile for each tumor type before delving into more specific lines of inquiry, the total number of differentially methylated regions (DMRs) was quantified. Our definition of a DMR includes surviving FDR correction (q < 0.01) and that the region must contain two or more CpGs to qualify. The total number of statistically significant differentially methylated regions between the TCGA cancer tissues and corresponding normal tissues utilized ranged from 3233 for esophageal squamous cell carcinoma (ESCA_SC) to 45,823 for kidney renal clear cell carcinoma (KIRC) (Fig. 1). While most cancer tissues showed a larger portion of hypermethylated DMRs in total, the bladder (BLCA), liver (LIHC), thyroid (THCA), and uterus (UCEC) cancers had a higher proportion of DMRs in the hypomethylated state (Fig. 1). The results of the chi-squared test revealed for the full hyper- and hypomethylation counts that there is a statistically distinct pattern between cancer types and that this pattern is both associated with and dependent on the cancer tissue (p < 0.001).

### Homeobox genes

**Figure 2:**
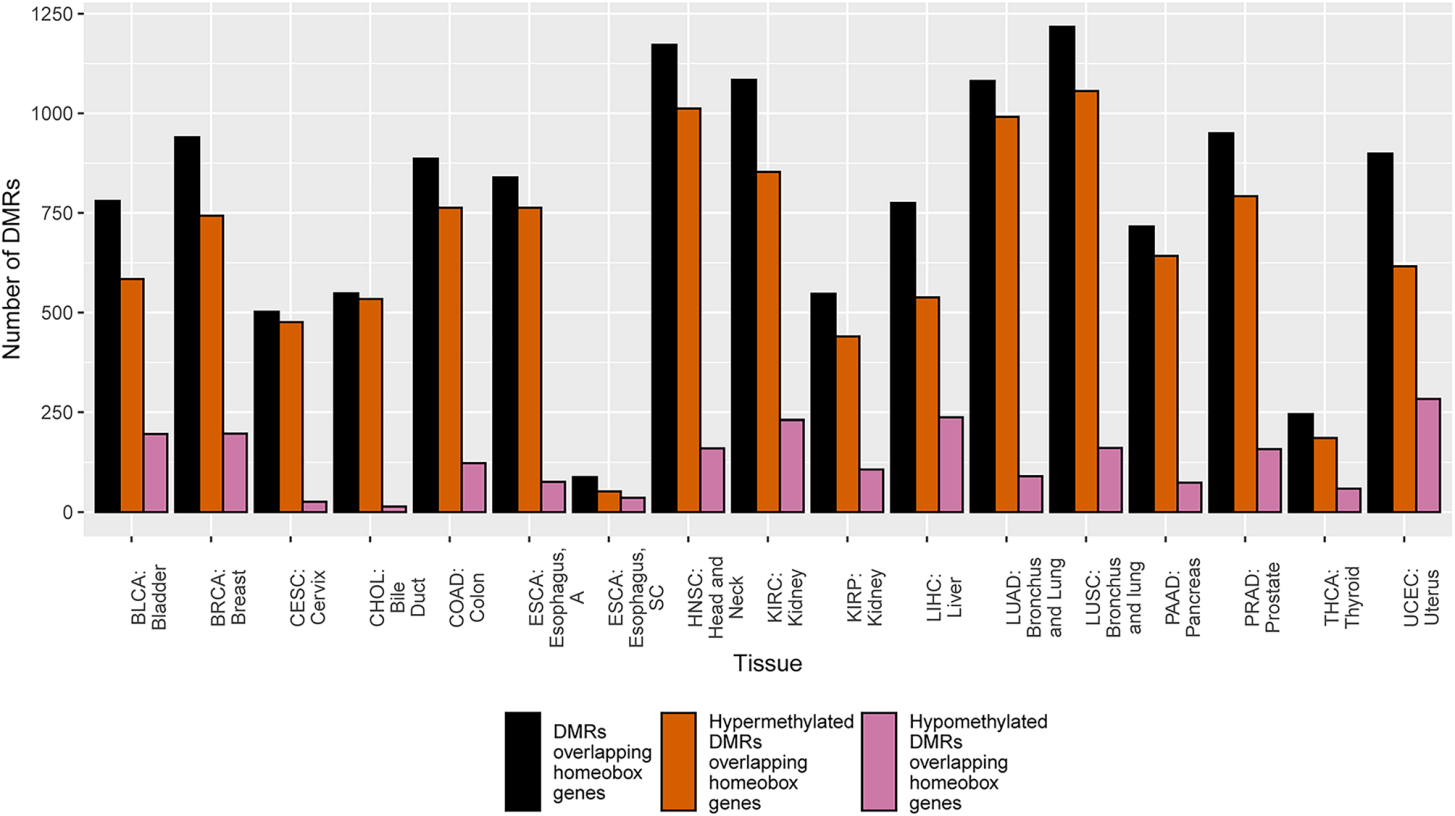
Hyper- and hypomethylated differentially methylated regions overlapping homeobox in the listed TCGA cancer type compared to total DMRs overlapping homeobox genes.

To specifically investigate the DNA methylation landscape of homeobox genes across different tumor types, the same definition of a DMR was used and the total DMRs were filtered to include only those that overlapped homeobox genes. Overall, only a small number of statistically significant hypermethylated DMRs overlapped with the list of queried homeobox genes from the literature as depicted in Fig. 2, the maximum number being 1056 from the lung squamous cell carcinoma (LUSC) dataset. Although this number is a small fraction of all DMRs, when we consider that there are only about 200 homeobox genes, the data indicate that each homeobox gene in LUSC contains 5 DMRs on average. There were only 52 homeobox DMRs in the esophageal squamous cell carcinoma (ESCA_SC) dataset. In cholangiocarcinoma (CHOL), 97.4% of all statistically significant DMRs overlapping homeobox genes were hypermethylated. Additionally, 76.6% of *total* DMRs in cholangiocarcinoma were hypermethylated, which is the highest value of all the TCGA cancer types based on this analysis. Overall, hypermethylation DMRs dominated in homeobox genes and hypomethylation DMRs were very rare (Fig. 1). Based on the performed permutation test, eleven cancer tissue types showed a representation of DMRs in homeobox genes greater than by random chance. These tumor sets include: both esophageal tumors (p < 0.001), uterine (p < 0.02), pancreas (p < 0.001), both lung (p < 0.001), head and neck (p < 0.001), bladder (p < 0.01), colon (p < 0.001), bile duct (p < 0.001), and cervical cancer tumors (p < 0.001). The results of our chi-squared test on this homeobox-specific count data revealed that the hypo- and hypermethylation frequencies are distinct statistically (p < 0.001) and that this profile is both associated with and dependent on cancer tissue type.

### Polycomb repressive complex 2 target genes

**Figure 3:**
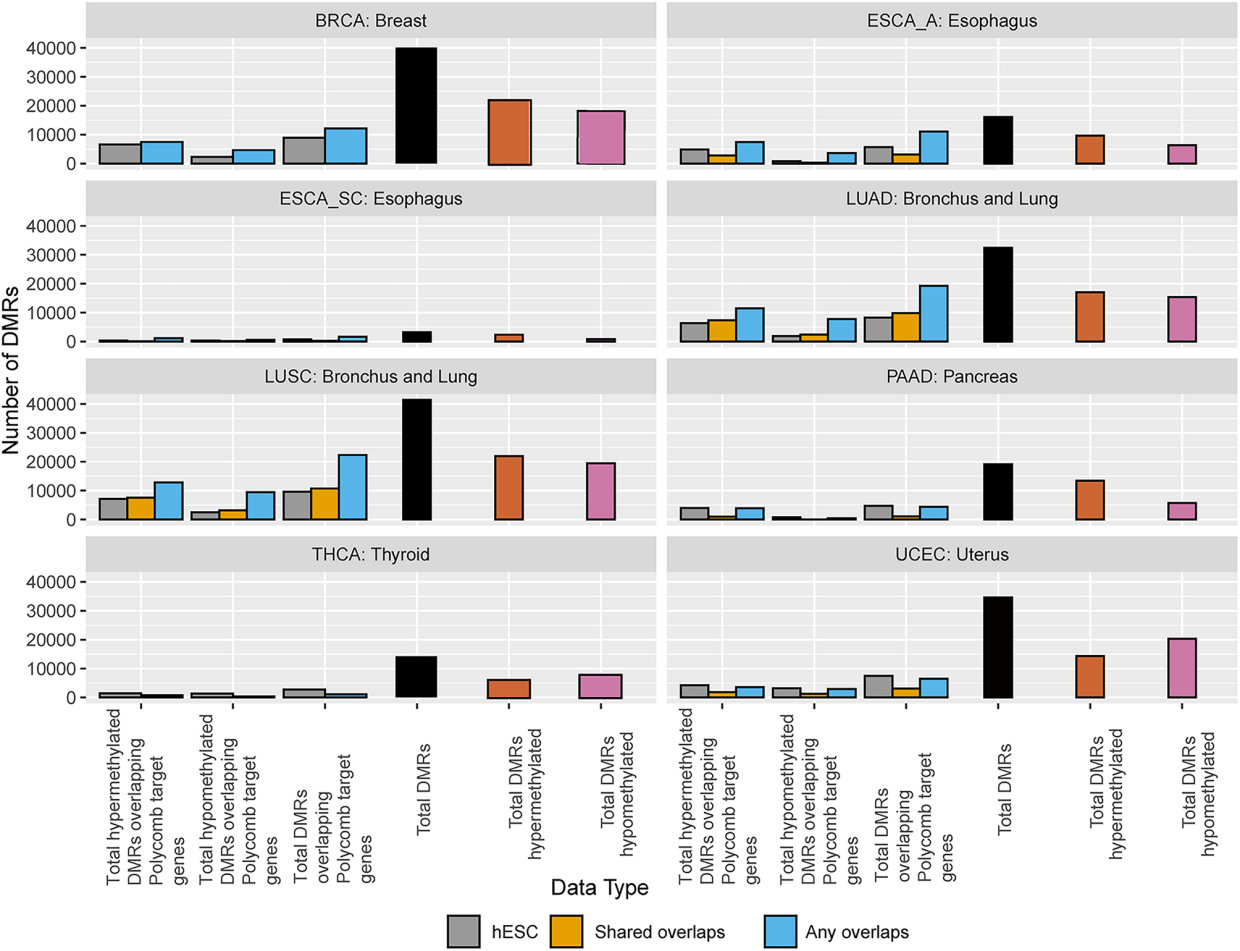
Number of statistically significant hyper- and hypomethylated DMRs for Polycomb repressive complex 2 target genes by TCGA cancer type. “Shared overlaps” is defined as the overlap between the DMRs and the H3K27me3 peaks present in all samples and “Any overlaps” means a given DMR overlaps with an H3K27me3 peak in one or more ChIP-seq samples. hESC represents the hESC H1 H3K27me3 data downloaded from GEO (GSM466734). The BRCA and THCA sets do not have a Shared overlaps category because there was only one normal tissue sample, so “Any overlaps” encompasses the data.

**Figure 4:**
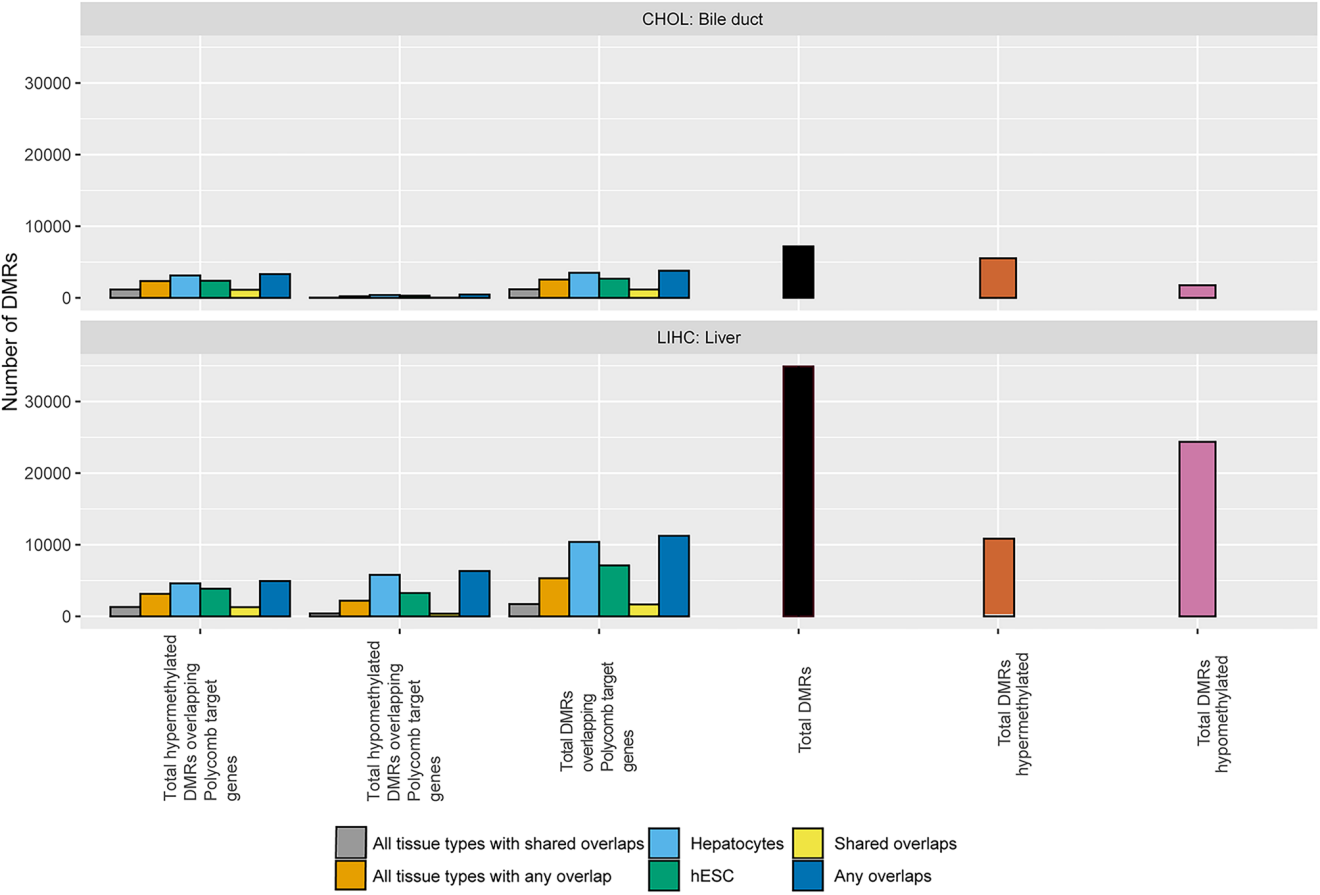
Number of hyper- and hypomethylated DMRs for Polycomb repressive complex 2 target genes for TCGA hepatic cancer types. “Shared overlaps” is defined as the overlap between the DMRs and the H3K27me3 peaks present in all samples and “Any overlaps” means a given DMR overlaps with an H3K27me3 peak in one or more ChIP-seq samples. hESC represents the H1 H3K27me3 data downloaded from GEO. “All tissue types with any overlap” indicates that the entirety of the DMRs were used, and ”All tissue types with shared overlap” indicates that only common DMRs were used.

A similar procedure as to what has been described previously was followed to interrogate Polycomb repressive complex 2 (PRC2) target genes, in which the overlap was taken between known PRC2 target sites and the genomic locations of our DMRs. In terms of the DMRs overlapping Polycomb repressive complex 2 target genes, typically, the matched tissues from the ENCODE database yielded a higher number of DMRs than the hESC H1 H3K27me3 data. In Fig. 3 “Shared overlaps” is defined as the overlap between the DMRs and the H3K27me3 peaks present in all samples and “Any overlaps” means a given DMR overlaps with an H3K27me3 peak in one or more ChIP-seq samples. Esophageal adenocarcinoma (ESCA) shows the highest percentage of hypermethylated Polycomb target gene DMRs among total hypermethylated DMRs at 76.3% in its “Any overlaps” category among all categories in all TCGA cancer types. In some cases, “Shared overlaps” fell below the hESC H3K27me3 data, indicating that the normal tissue samples perhaps differ from each other more than they share commonalities. Figure 4 displays the same information as Fig. 3, except for only hepatic cancers due to the inclusion of hepatocytes as a primary cell type. The “Any overlaps” and “All tissue types with any overlaps” had the largest overlap of DMRs and Polycomb target genes, mirroring the pattern seen in Fig. 3 (Fig. 4).

### The overlap of differentially methylated homeobox and PRC2 target genes

Similarly, the method in which we queried PRC2 overlap with homeobox genes follows the same general procedure in which the overlaps of the genomic regions were cross referenced with known PRC2 target sites. The DMRs overlapping homeobox genes overlap more H3K27me3 peaks than by random chance (Supplementary Table 1). In Supplementary Table 2, the number of statistically significant differentially methylated homeobox genes that are also differentially methylated Polycomb target genes are listed, including the percentage of homeobox genes also covered by the PRC2 mark. Overall, cholangiocarcinoma showed the highest percentage of overlap of PRC2 target genes and homeobox genes at 98% for “any overlaps” and the hepatocytes, with the total primary tissue coming in at 94.5% (Supplementary Table 2). Esophageal adenocarcinoma followed at 94.7% for “Any overlaps”. The lowest crossover between the PRC2 and homeobox genes was the shared overlaps of tissues in esophageal squamous cell carcinoma. The data indicate that most homeobox genes are Polycomb targets.

### Sets of hyper- and hypomethylated DMRs in homeobox genes

**Figure 5:**
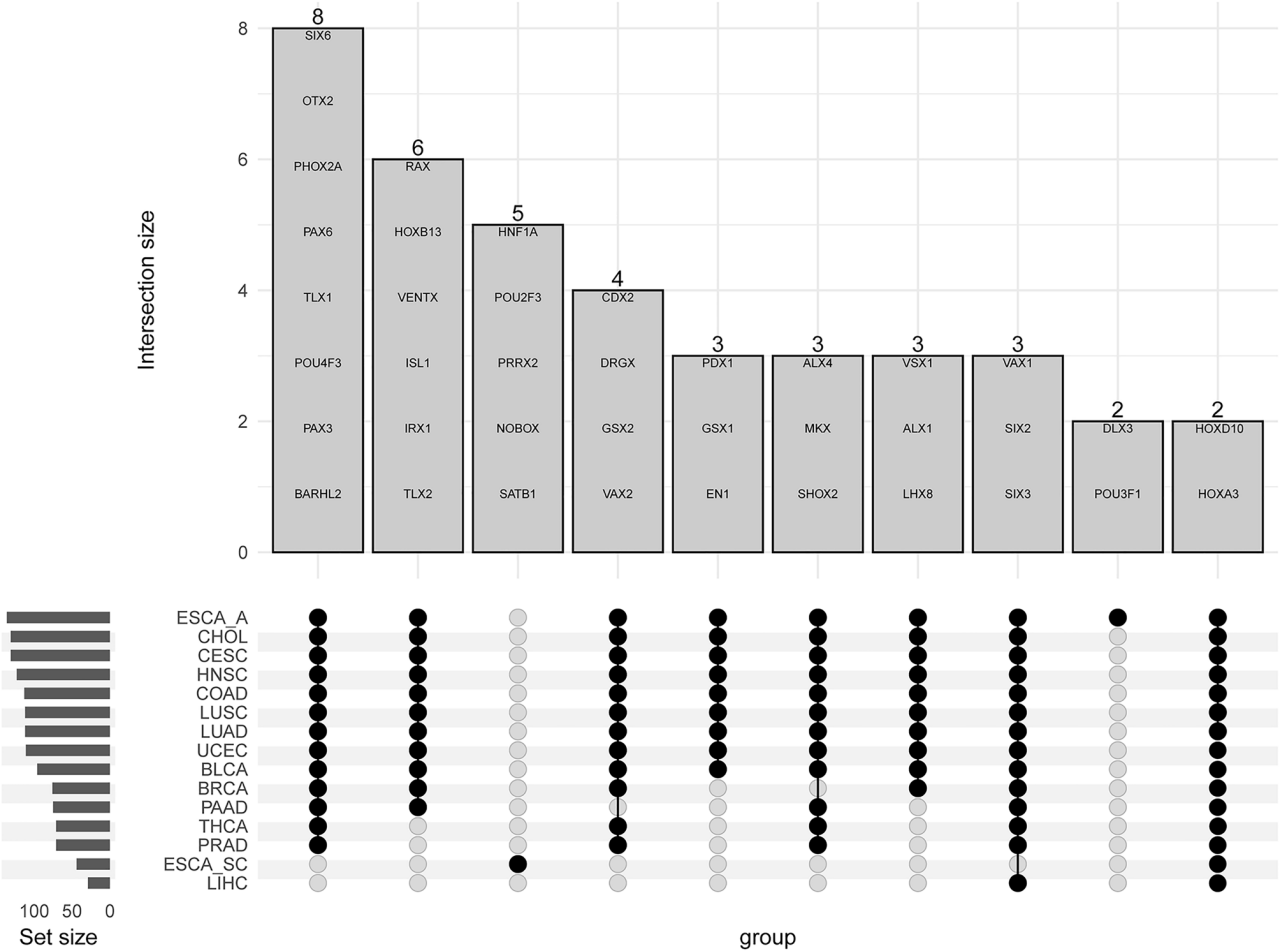
An upset plot^42,43^ showing the first 10 intersections between the TCGA cancer types and hypermethylated DMRs overlapping homeobox genes that met the change of 0.2 or greater in the beta values. The homeobox genes within the intersection set are listed within the bar plot. A black dot on the intersection portion of the figure indicates that the cancer type contains the hypermethylated genes within the bars. The set size represents the number of hypermethylated homeobox gene DMRs associated with the TCGA cancer type.

Focusing on the statistically significant hypermethylated DMRs overlapping homeobox genes, the set of homeobox genes hypermethylated in all 16 TCGA cancer types were the *HOXD10* and *HOXA3* genes. From the results in Fig. 5, Table 3 was created to show the TCGA cancer types that demonstrate representation of only a single hypermethylated homeobox gene not found in other cancers, indicating a possible cancer-specific biomarker. We define a biomarker as being a statistically significant DMR after FDR correction that has a greater than 0.2 change in the beta values between normal and tumor tissue^41^ and is found in only one tumor tissue type. In total, seven different cancer types displayed unique hypermethylated homeobox genes. Curiously, esophageal squamous cell carcinoma yielded the highest amount of potential hypermethylated biomarkers (Table 3) while having the smallest number of DMRs, both hyper- and hypomethylated (Figs. 1 and 2).

**Table 3:** Hypermethylated DMRs overlapping homeobox genes that are only found in one TCGA cancer type.

TCGA cancer	Gene
CHOL	*CERS2*
COAD	*ZEB1*
ESCA adenocarcinoma	*POU3F1, DLX3*
ESCA squamous cell	*SATB1, NOBOX, PRRX2, POU2F3, HNF1A*
KIRP	*SIX5*
LIHC	*PBX4*
UCEC	*CERS3, TGIF1*

The DMRs must have a greater than 0.2 difference in the beta value between tumor and normal tissue to meet this classification.

**Figure 6:**
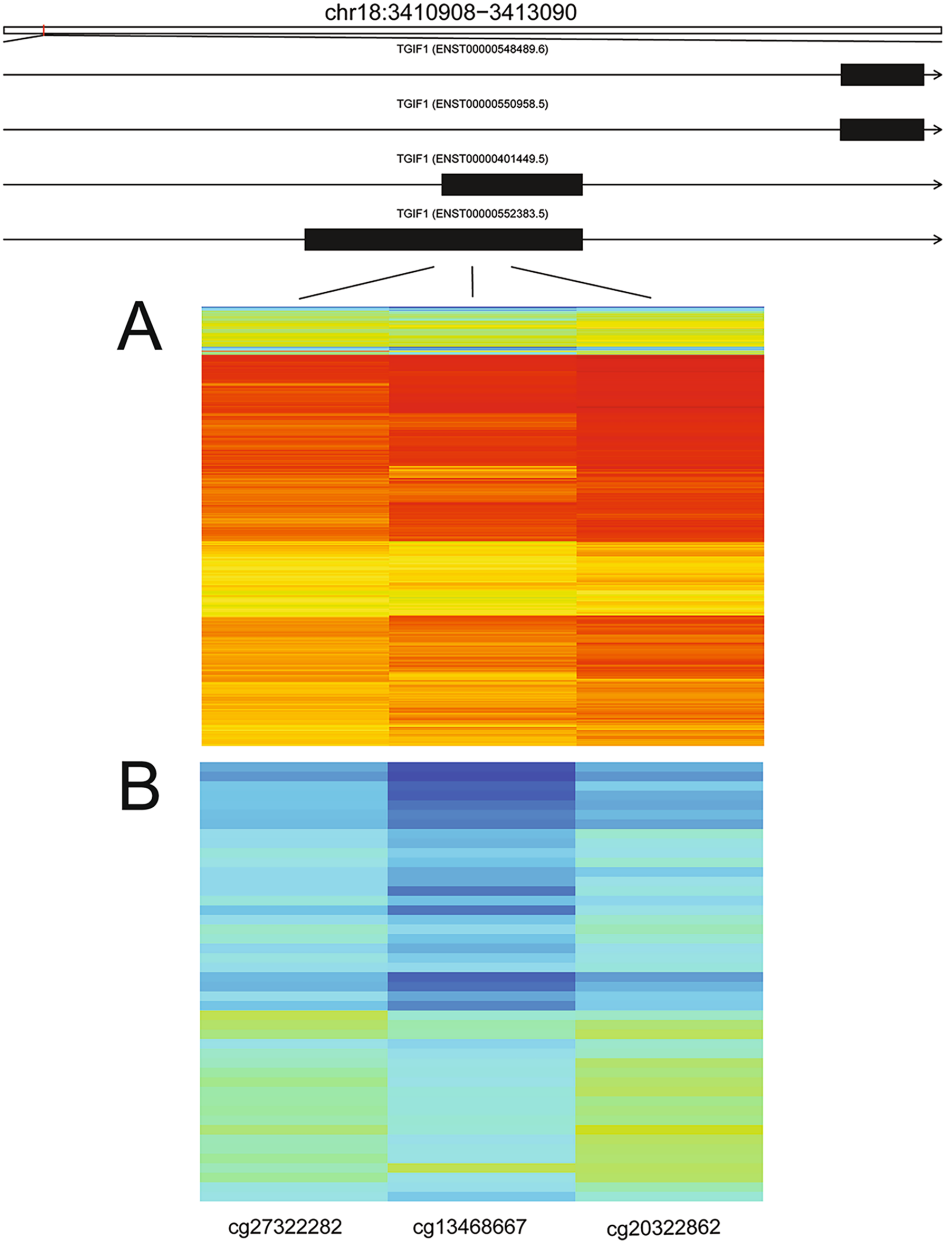
A heatmap of a statistically significant DMR meeting our definition of a biomarker in the UCEC dataset using the hg38 assembly. The hypermethylated DMR overlaps the *TGIF1* gene exons, represented as the black boxes which vary in genome position based on transcript isoform, and is composed of three probes. (**A**) The tumor samples (n = 438), and (**B**) Normal tissue (n = 46). The gradient of color goes from red to blue indicating hypermethylation to hypomethylation.

To substantiate our results, the hypermethylated potential biomarker *TGIF1* in the UCEC dataset was examined. The statistically significant DMR which also met the requirement of a greater than 0.2 difference in the change of the beta values between tumor and normal tissue was delineated and the beta values for the three probes encompassing the DMR were examined in a heatmap (Fig. 6). As seen in Fig. 6, the heatmap shows hypermethylation in tumor tissue samples (A) and hypomethylation in the normal tissue samples (B), which supports our determination of *TGIF1* as a hypermethylated biomarker in uterine cancer.

**Figure 7:**
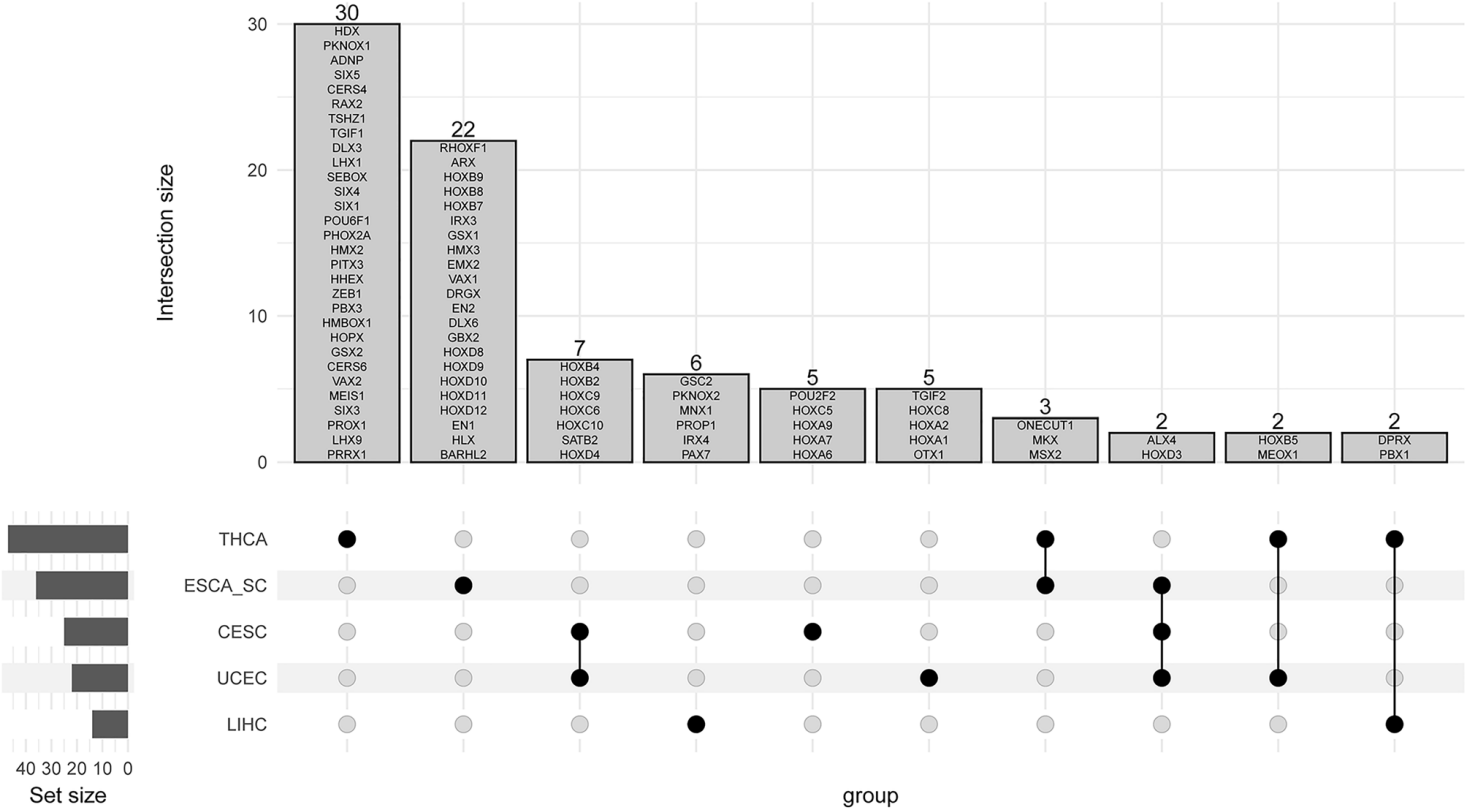
An upset plot^42,43^ showing the first 10 intersections between the TCGA cancer types and hypomethylated DMRs overlapping homeobox genes that met the change of 0.2 or greater in the beta values. The homeobox genes within the intersection set are listed within the bar plot. A black dot on the intersection portion of the figure indicates that the cancer type contains the hypomethylated genes within the bars. The set size represents the number of hypomethylated homeobox gene DMRs associated with the TCGA cancer type.

The same process was repeated for hypomethylated DMRs overlapping homeobox genes. In this case, there was less overlap between the cancer types, as indicated by Table 4, which is representative of Fig. 7. There were no homeobox genes that contained a hypomethylated DMR that encompassed the entire set of tumor types. ESCA_SC and THCA had comparatively fewer DMRs overall than other tumor tissue types (Figs. 1 and 2); however, 30 DMRs overlapped homeobox genes that met our definition of a biomarker for the thyroid and 22 for esophageal squamous cell carcinoma (Fig. 7; Table 4).

**Table 4:** Hypomethylated DMRs overlapping homeobox genes that are only found in one TCGA cancer type.

TCGA cancer	Gene
BLCA	*ISL1, NOBOX*
CESC	*HOXA6, HOXA7, HOXA9, HOXC5, POU2F2*
CHOL	*MEIS2*
ESCA squamous cell	*BARHL2, HLX, EN1, HOXD12, HOXD11, HOXD10, HOXD9, HOXD8, GBX2, DLX6, EN2, DRGX, VAX1, EMX2, HMX3, GSX1, IRX3, HOXB7, HOXB8, HOXB9, ARX, RHOXF1*
LIHC	*PAX7, IRX4, PROP1, MNX1, PKNOX2, GSC2*
THCA	*PRRX1, LHX9, PROX1, SIX3, MEIS1, VAX2, CERS6, GSX2, HOPX, HMBOX1, PBX3, ZEB1, HHEX, PITX3, HMX2, PHOX2A, POU6F1, SIX1, SIX4, SEBOX, LHX1, DLX3, TGIF1, TSHZ1, RAX2, CERS4, SIX5, ADNP, PKNOX1, HDX*
UCEC	*TGIF2, OTX1, HOXA1, HOXA2, HOXC8*

The DMRs must have a greater than 0.2 difference in the beta value between tumor and normal tissue to meet this classification.

## Discussion

The current work is, to our knowledge, the first pan-cancer analysis that delves into the differential methylation status of homeobox genes, Polycomb repressive complex 2 (PRC2) target genes, and the intersection between the two. Overall, our study suggests a small overlap between statistically significant hypermethylated differentially methylated regions (DMRs) and homeobox genes, but this is largely due to the small number of homeobox genes in the genome (~ 200). We demonstrated unique patterns in hyper- and hypomethylated DMRs overlapping homeobox genes, including the potential identification of biomarkers via our upset plots (Figs. 5 and 7). Interestingly, only seven homeobox genes out of the queried 206 did not contain a DMR, neither hypo- nor hypermethylated. These genes include *HMX1*, *NANOG*, *LEUTX*, *SHOX*, *TGIF2LX*, *RHOXF2B* and *RHOXF2*.

Cancers acquire global DNA hypomethylation in the genome with hypermethylation occurring most prominently at CpG islands (reviewed in Ehrlich, 2009 ^44^), of which 96% are covered on the Illumina HM450k array^30^. Curiously, the THCA, UCEC, BLCA, and LIHC showed higher DMR hypomethylation frequencies globally based on our DMR definition in Fig. 1, which was unexpected. Though CpG islands contain this characteristic focal hypermethylation, it is possible that in these cases we see the greater hypomethylation based upon our 2-loci definition of a DMR. The Illumina HM450k array has approximately 9.92 loci per gene body and 5.63 loci per CpG island^30^. Based on our definition of a DMR, it appears more likely that the hypomethylated gene body would then be weighted more heavily. Why this pattern is present in only four of the TCGA datasets is unknown.

As previously stated, homeobox genes are well known PRC2 target genes. Published research in breast cancer has shown that 53% of hypermethylated genes are known Polycomb targets which includes the homeobox gene family^20^. Similar results have been shown in lung cancer^16^. Our results are intriguing in that our reported overlap between homeobox genes and PRC2 target genes are much higher than previously appreciated. However, our results include existence of both hyper- and hypomethylated DMRs at homeobox genes, although hypermethylated DMRs are much more common based on overall spread of the homeobox genes and the number that are shared between tumor types (Fig. 2; Figs. 5 and 7).

While past studies have focused on the DNA methylation of a single gene in some specific cancers or focused on DNA methylation sites shared across cancer types this study profiles a large group of cancers to look at global DNA methylation characteristics before investigating a specific gene family that lacks major representation in the literature. Methods for other pan-methylome analyses have used linear models to investigate a combined set of cancers collectively^45^ with the aim of diagnosing metastatic cancers of unknown origin. This approach however relied on “epi-driver” genes which are defined as an “aberrantly expressed gene in cancers”^46^ and lack mechanistic specificity. Interestingly, the work by Liu and colleagues^47^ used a more stringent definition of a DMR than the current study (10 CpGs instead of 2). However, a main finding from that study was the differential methylation of the *HOXA* locus, which the current study also reports in the shared hypermethylation of *HOXA3* across cancers. The present study additionally separated DMRs into hypo- and hypermethylated regions whereas Liu and colleagues only required differential methylation which would result in a larger pool of regions. Finally, our study lacks integration of the results with expression data as presented in the works of those such as Saghafinia et al.^48^, however the stringency and classification of our DMRs, specifically the breakdown into hyper- and hypomethylation and our investigation into an underexplored gene class lends weight to the present study and distinguishes it from previous works in the literature.

From the intersection of the TCGA tumor types with homeobox genes, hypermethylation of *CERS2* was identified as a biomarker for the cholangiocarcinoma (CHOL) dataset, functioning as a tumor suppressor^49,50^. Our definition of a biomarker is a hypo- or hypermethylated DMR that overlaps a homeobox gene in a single tumor dataset that also passes the set threshold of a difference of 0.2 in the beta values between normal and tumor tissue samples in that statistically significant DMR.

Esophageal adenocarcinoma displayed two hypermethylated biomarkers, one being *POU3F1*, whose methylation status has already been shown to function successfully as a biomarker^51^. The frequency with which our identified biomarkers are supported in the literature lends confidence to our analyses and provides support for the biomarkers identified that show a reduced presence in previously published studies. Esophageal squamous cell carcinoma hypermethylated DMRs overlapping homeobox genes yielded five potential biomarkers. Two of these markers, *NOBOX and PRRX2* show no representation in the literature in association with this cancer type. Conversely, *SATB1* has been shown in association with esophageal squamous cell carcinomas through an overexpression^52^. Finally, *HNF1A* has been associated with the epithelial-to-mesenchymal transition in esophageal cancers, and its hypermethylation^53^ contributes to its malignant progression.

Continuing with hypermethylated DMRs overlapping homeobox genes in a single tumor tissue that also meets our set threshold, the KIRP, or kidney renal papillary cell carcinoma, dataset yielded *SIX5* as a potential biomarker. Liver hepatocellular carcinoma (LIHC) also yielded a biomarker, the *PBX4* gene^54^, which has shown to be hypermethylated in this cancer type^55^. Finally, the UCEC dataset focusing on corpus endometrial carcinomas in the uterus showed hypermethylated DMRs overlapping the *CERS3* and *TGIF1* homeobox genes. *CERS3* is a signature gene in cervical cancer^56,57^, but has no current relationship to uterine cancer, and *TGIF1* additionally is considered a significantly mutated gene in cervical cancer^58^. Interestingly, *TGIF2* was shown to have an overlapping hypomethylated DMR in the UCEC dataset. Though a paralog with *TGIF1* which showed hypermethylation in UCEC*,* the two genes do not share similarities in terms of function. However, *TGIF2* is also heavily associated with cervical cancer metastasis^59^.

Of note, the *HOXD10* and *HOXA3* homeobox genes showed a hypermethylated DMR with a greater than 0.2 change in the beta value in every tumor tissue type in the present study. *HOXD10* is a tumor suppressor involved in the malignancies of many different tissue types. Its hypermethylation across the tissues lends support to our hypotheses. *HOXD10* and *HOXA3* are both targets of the miR-10 family^60^. *HOXA3*, however, does not have a pervasive presence in the literature across cancer types and thus could potentially be a finding that could be used in a general cancer screening.

In the bladder cancer dataset, *ISL1* was selected as a hypomethylated biomarker. *ISL1* methylation has been shown previously to function as a biomarker for bladder cancer^61^ and is heavily associated with the cancer type^62^ as developmentally it is involved in forming the urinary tract^63^.

Higher expression of the *HOXA* family of transcription factors has previously been proposed as a prognostic marker for cervical cancer^64^. Here we show the presence of *HOXA6, HOXA7,* and *HOXA9* as meeting our definition for hypomethylated biomarkers in the TCGA cervical cancer tumor dataset. Finally, *POU2F2* has been considered to be a new potential driver^65^ for cervical cancer and was an additional finding from the CESC dataset.

In hypomethylated DMRs overlapping homeobox genes meeting our definition of a potential biomarker, esophageal squamous cell carcinoma yielded 22 different genes. These genes contain DMRs defined by the presence of two or more probes, that have a greater than 0.2 difference in beta values between normal and tumor tissue methylation, and are statistically significant after FDR correction, so it was surprising to find so many. Those of note include those in the *HOXD* gene cluster, which contributed five genes to this list. This cluster has been shown to be heavily involved in cancer (reviewed in Wang et al.^66^) but never directly linked to the esophagus. Similarly, *HOXB* cluster dysregulation, and dysregulation of the HOX genes in general, are closely linked to cancer, though these specific genes (*HOXB7, HOXB8, and HOXB9)* have not been shown in specific relation to esophageal squamous cell carcinomas. *EN1*, however, was identified in a study focusing on esophageal squamous cell carcinomas as being differentially methylated. ^67^ Similarly, *EN2* is upregulated in esophageal squamous cell carcinomas^68^.The genes that have no apparent relationship with esophageal squamous cell carcinomas include *BARHL2, HLX, DLX6, DRGX, HMX3, GSX1, IRX3, ARX,* and *RHOXF1.*

Thyroid carcinomas (THCA) produced 30 hypomethylated DMRs overlapping homeobox genes meeting our biomarker definition. The homeobox gene *PRRX1* has been defined as a transition marker for the epithelial-to-mesenchymal transition^69^ in thyroid carcinomas. The *SIX* family of transcription factors (*SIX1, SIX3, SIX4, SIX5),* were prominent in the THCA dataset in the list of potential hypomethylated biomarkers. *SIX1* has been shown to be upregulated in thyroid cancers and associated with tumor size and metastasis^70^. Papillary thyroid carcinomas show an upregulation in *VAX2*^71^, and its development is facilitated by *CERS6*^72^*. HHEX* is considered a tumor suppressor gene in the thyroid and plays a crucial role in development of the gland^73^. Metastasis to the lymph nodes from thyroid carcinomas has been demonstrated to be influenced by *LHX1* upregulation^74^. Like *ZEB1*, *TGIF1* was a potential hypermethylated biomarker identified in another tumor tissue type in this analysis. In thyroid cancer, *TGIF1* is upregulated in the papillary subtype, which aligns with our observation of hypomethylation in a DMR in the gene^75^. We show that *CERS4* is differentially methylated in thyroid cancer, with evidence from the literature that it is also differentially expressed^76^. Finally, *PKNOX1* has been identified as a key component in angiogenesis in thyroid carcinomas^77^. The genes from our potential hypomethylated biomarkers that have no reported relationship with thyroid cancer include *LHX9, MEIS1, GSX2, PITX3, HMX2, PHOX2A, POU6F1, SEBOX, DLX3, TSHZ1, RAX2, ADNP,* and *HDX.*

The thyroid tumor type had the most potential biomarkers in the hypomethylated state. Interestingly, the THCA dataset also had the lowest overlap in homeobox genes and PRC2 target genes. It has been shown previously that genes that have hypermethylated CpG islands that are also PRC2 targets are more plastic in their regulatory mechanisms, showing variability in methylation status and expression among different cancer types^78^.

The last tumor tissue type to have potential hypomethylated biomarkers called was the uterine tissue (UCEC). These included *OTX1,* which is important in endometrial cancers and shows differential methylation between early- and late-onset^79^, *HOXA1* and *2,* in which *HOXA1* is a known oncogene in mammary tissue^80^. *HOXA2* is differentially methylated in endometriosis^81^. *HOXC8* similarly is differentially methylated in endometriosis^81^, while *TGIF2* is expressed in ovarian cancers^82^.

Limitations to the present study include the utilization of multiple different cancer subtypes within the TCGA dataset. For example, the BRCA data includes twenty different subtypes of breast cancer, which were not narrowed down to preserve our statistical power. However, datasets such as LUSC and LUAD split the cancer into distinct subtypes (in this case lung adeno- and squamous cell carcinoma) and datasets such as esophageal cancer (ESCA) were divided into adeno- and squamous cell carcinomas. The specific tissues used in PRC2 target site identification may also have yielded results that would have been improved by a higher sample size or a more specific cell type if available. Curiously, in many cases the overlap of the tissues resulted in less target sites than the unspecific hESC data and showed a large disparity between the total tissues used and the overlap. This indicates that the normal tissues were quite dissimilar to each other and could potentially function as a confounder. However, using ESC chromatin marks is a valid approach because the majority of regions occupied by PRC2 in ESCs retain the H3K27me3 across differentiated cell types of all lineages^38^. Finally, though viewed as a strength by our research team, the definition of a DMR based on two or more CpG sites would reduce the results obtained as opposed to simply including the statistically significant DMRs called by our bioinformatics that only contained one CpG. This, however, increases our confidence in the presence and accuracy of a DMR definition.

In conclusion, this study provides novel information about the differential methylation across and between cancer types, with an emphasis on homeobox genes, PRC2 target genes, and their intersection. By comparing our results to the existing cancer literature, we have both confirmed the status of homeobox gene methylation as useful biomarkers, and proposed new genes that could be potentially investigated and confirmed as possible cancer-specific biomarkers, even for seemingly unrelated tissue types. Aside from screening methods incorporating our biomarkers for the presence of cancer, the results from this study could also be utilized in classifying tumors with an unknown primary site. It is estimated that up to 5% of all new cancer diagnoses are from metastatic cancer with an unknown primary site, resulting in it being the third to fourth most common cause of cancer-related death^83^. It is suggested that biomarker results from this study could be verified in vitro or in vivo or repeated using a more thorough methylation array such as the EPIC array or using WGBS to find more targets and provide increased accuracy in calculation of the global hyper- and hypomethylation events.

### Supplementary Information

Supplementary Table 1.

Supplementary Table 2.

## Data Availability

The datasets analyzed during the current study are available in TCGA (https://portal.gdc.cancer.gov/analysis_page?app=Downloads) and ENCODE (https://www.encodeproject.org), with all identifiers for datasets included in the Methods section and in Table 2.
