# Supplementary material for: Homeobox and Polycomb target gene methylation in human solid tumors
Source: Sci Rep. 2024 Jun 17;14:13912. doi: 10.1038/s41598-024-64569-5 (PMC11183203; doi:10.1038/s41598-024-64569-5)
Supplement: Supplementary file 1 — Supplementary Table 1. [file 41598_2024_64569_MOESM1_ESM.docx]

| Tissue | p-value | Z-score |
| --- | --- | --- |
| ESCA_S | < 0.001 | 5.4642 |
| ESCA_A | < 0.001 | 21.8111 |
| UCEC | < 0.001 | 14.8642 |
| THCA | 0.015 | 2.7384 |
| PRAD | < 0.001 | 11.8097 |
| PAAD | < 0.001 | 16.0283 |
| LUSC | < 0.001 | 16.1925 |
| LUAD | < 0.001 | 15.8037 |
| LIHC | < 0.001 | 12.0465 |
| KIRP | < 0.001 | 8.5737 |
| KIRC | < 0.001 | 12.473 |
| HNSC | < 0.001 | 19.2974 |
| COAD | < 0.001 | 19.6263 |
| CHOL | < 0.001 | 14.0741 |
| CESC | < 0.001 | 19.8564 |
| BRCA | < 0.001 | 12.2593 |
| BLCA | < 0.001 | 14.8445 |

**Supplementary Table 1. The p-value and Z-scores by cancer tissue type from the results of a permutation test.** All of the tissues show statistical significance, indicating that the DMRs overlapping homeobox genes statistically have more H3K27me3 than by random chance.
