# Supplementary material for: Homeobox and Polycomb target gene methylation in human solid tumors
Source: Sci Rep. 2024 Jun 17;14:13912. doi: 10.1038/s41598-024-64569-5 (PMC11183203; doi:10.1038/s41598-024-64569-5)
Supplement: Supplementary file 2 — Supplementary Table 2. [file 41598_2024_64569_MOESM2_ESM.docx]

| TCGA Cancer | Tissue type | DMRs overlapping both homeobox genes and Polycomb target genes | Percentage of homeobox genes overlapped by a Polycomb target gene DMR |
| --- | --- | --- | --- |
| BLCA | hESC | 517 | 66.2% |
| BRCA | hESC | 595 | 63.2% |
|  | Any overlaps | 719 | 76.5% |
| CESC | hESC | 421 | 83.8% |
| CHOL | Shared overlaps | 420 | 74.6% |
|  | Any overlaps | 409 | 98% |
|  | Hepatocytes | 537 | 98% |
|  | All tissue types shared overlaps | 537 | 74.6% |
|  | All tissue types with any overlaps | 409 | 94.5% |
| COAD | hESC | 518 | 72.5% |
| ESCA, adenocarcinoma | hESC | 645 | 76.9% |
|  | Shared overlaps | 660 | 78.6% |
|  | Any overlaps | 795 | 94.7% |
| ESCA, squamous cell carcinoma | hESC | 57 | 64.8% |
|  | Shared overlaps | 48 | 54.5% |
|  | Any overlaps | 73 | 83% |
| HNSC | hESC | 846 | 72.2% |
| KIRC | hESC | 725 | 66.9% |
| KIRP | hESC | 305 | 55.7% |
| LIHC | hESC | 486 | 62.7% |
|  | Shared overlaps | 440 | 56.8% |
|  | Any overlaps | 657 | 84.8% |
|  | Hepatocytes | 656 | 84.6% |
|  | All tissue types shared overlaps | 440 | 56.8% |
|  | All tissue types with any overlaps | 563 | 72.6% |
| LUAD | hESC | 782 | 72.3% |
|  | Shared overlaps | 969 | 89.6% |
|  | All overlaps | 1013 | 93.7% |
| LUSC | hESC | 837 | 68.8% |
|  | Shared overlaps | 1052 | 86.4% |
|  | All overlaps | 1118 | 91.9% |
| PAAD | hESC | 492 | 68.7% |
|  | Shared overlaps | 415 | 58% |
|  | All overlaps | 615 | 86% |
| PRAD | hESC | 614 | 64.6% |
| THCA | hESC | 144 | 58.8% |
|  | All overlaps | 153 | 62.4% |
| UCEC | hESC | 612 | 68.1% |
|  | Shared overlaps | 535 | 59.5% |
|  | All overlaps | 638 | 71% |

**Supplementary Table 2. The number of differentially methylated regions overlapping both Polycomb target genes and homeobox genes by cancer and tissue type.** Also included is the percentage of identified differentially methylated homeobox genes that are also overlapped by a Polycomb target DMR.
